# Long Distance Dispersal Potential of Two Seagrasses *Thalassia hemprichii* and *Halophila ovalis*

**DOI:** 10.1371/journal.pone.0156585

**Published:** 2016-06-01

**Authors:** Kuoyan Wu, Ching-Nen Nathan Chen, Keryea Soong

**Affiliations:** 1 Department of Oceanography, National Sun Yat-sen University, Kaohsiung, Taiwan; 2 Asia-Pacific Ocean Research Center, National Sun Yat-sen University, Kaohsiung, Taiwan; United States Department of Agriculture, UNITED STATES

## Abstract

The wide distribution of many seagrasses may be attributable to exploitation of currents. However, many species have seeds heavier than seawater, limiting surface floating, and thus, deep water becomes a potential barrier between suitable habitats. In this investigation, we studied the dispersal potential of various life history stages of two species of seagrasses, *Thalassia hemprichii* and *Halophila ovalis*, at Dongsha Atoll and Penghu Islands in Taiwan Strait, west Pacific. The adult plants of both species, often dislodged naturally from substrate by waves, could float, but only that of *T*. *hemprichii* could float for months and still remain alive and potentially able to colonize new territories. The seedlings of *T*. *hemprichii* could also float for about a month once failing to anchor to substrate of coral sand, but that of *H*. *ovalis* could not. The fruits and seeds of *T*. *hemprichii* could both float, but for too short a duration to enable long distance travel; those seeds released from long floating fruits had low germination rates in our tests. Obviously, their seeds are not adaptive for long distance dispersal. Fruits and seeds of *H*. *ovalis* do not float. The potential of animals as vectors was tested by feeding fruits and seeds of both species to a goose, a duck, and two fish in the laboratory. The fruits and seeds of *T*. *hemprichii* were digested and could no longer germinate; those of *H*. *ovalis* could pass through the digestive tracts and have a much higher germination rates than uningested controls. Therefore, birds could be important vectors for long distance dispersal of *H*. *ovalis*. The two seagrasses adopted very different dispersal mechanisms for long distance travel, and both exploited traits originally adaptive for other purposes.

## Introduction

Dispersal is one of the fundamental traits essential for all species [1]. Selective pressures on different levels, *e*.*g*., individuals to populations, could often be alleviated by moving elsewhere. For example, scattered trees in the tropics may be less likely to suffer from predators/pathogens associated with parents [2,3]. Competition pressure from siblings and parents could also be reduced if dispersed at birth [4]. On a long time scale, suitable habitats could not last forever, and thus moving actively or passively is necessary for survival of genes and the species [5]. The extinction risks could perceivably be lessened with wide geographic distribution, especially if local extinction is frequent, *e*.*g*., in a dynamic environment in shallow waters.

The mechanism to achieve dispersal is highly diverse among species. Most terrestrial animals are mobile and can move more or less at will. Other organisms, especially plants, have to rely on different means. Wind and water as media can carry some plant propagules for long distances [6,7]. Many plants are dispersed passively by animals [8–10]. All these mechanisms could result in effective dispersal and wide distribution of a species [11], although we are not always sure which stage of life history contributes to the greatest extent of dispersal of a species.

For species exploring new habitats, e.g., from terrestrial to marine environments, dispersal could either become constrained in the new environment, or could use the opportunity available in the new environment for expanding ranges. Most seagrasses originated from terrestrial monocotyledons about 80–100 million years ago [12]. To them, currents in the sea may provide a great opportunity for dispersal if the organisms can exploit it [13–16], either by existing mechanisms or by new adaptations. This is especially true for seagrasses that can only survive in shallow waters with ample light. To reach suitable habitats, which are often widely scattered and discretely distributed, seagrass propagules have to cross open seas. In a study of the turtle grass, *Thalassia hemprichii*, it was found that their non-dormant seeds are adapted to grasping carbonate sand substrate as soon as possible. At least four novel traits of their seeds, *i*.*e*., shape of seeds, unipolar distribution of starch granules, growth of root-hair-like filaments on the basal surface of seeds, and sensitivity to blue light, were identified to help them secure quickly to the bottom in a wavy environment [17]. The disadvantage is that the dispersal potential of their seeds is thus very limited.

A similar situation of lack of dispersal stage occurs in *Halophila* spp., which has flowers/fruits/seeds all buried in the sediment. Their seeds can remain dormant for a long time, and germinate only when the sediment is disturbed and the seeds exposed to light [18]. Thus, temporal dispersal is likely the function of their underground seed bank. The heavier-than-water specific weight of their seeds is not helpful from the perspective of long distance spatial dispersal.

On the other hand, both species are known to have wide distribution in Indo-West Pacific (IUCN Red List). If their seeds do not have the functions of spatial dispersal, how do they accomplish their wide distributions?

Water is used by some land plants and mangrove trees as a dispersal medium to transport floating fruits, seeds, embryos, and even vegetative fragments [19]. It is likely that the same may apply to seagrasses [9,10,20,21]. Animals may also act as vectors carrying seagrass fruits or seeds, especially when seed sizes are small [22–24]. In this study, we studied four stages, *i*.*e*., fruits, seeds, seedlings and vegetative fragments, of adult plants of the two seagrass species to assess which stage had the greatest potential for long distance dispersal by either biotic or abiotic means.

## Materials and Methods

### Ethics statement

Collection permits at Dongsha Atoll National Park were granted by Marine National Park Headquarters of Taiwan. Permits for animal experiments were granted by the Animal Protection Committee of National Sun Yat-sen University, Taiwan.

### Seagrass sampling

Samples of various stages of *Thalassia hemprichii* were collected from Dongsha Island (N 20°43’, E 116°43’), and that of *Halophila ovalis* from Penghu Islands (N 23°38’, E 119°36’). The fruiting season of *T*. *hemprichii* at Dongsha Island occurs between January to March (personal observation), whereas *H*. *ovalis* has flowers and fruits in the sediment in spring and summer, the seasons of surveys at Dongsha [25]. A seed bank of *H*. *ovalis* exists in undisturbed sediment, in which seeds accumulated through the years [26].

### Floatation of fruits

The first batch of fruits of *T*. *hemprichii* was collected a few meters from the water line on the beach of Dongsha Island in January 2011. Since the beach was searched for the fruits every week, most of those collected are supposed to be on the beach within a week. A second batch of fruits were collected from piles of seagrass debris on the beach of Dongsha Island between February 6 and 20, 2014. They were more or less buried in the debris. Since the seagrass piles occurred seasonally, these seeds were produced within a few weeks. The floatation duration of the fruits was examined in plastic containers (45 x 30 cm) with aeration and daily seawater change.

*Halophila ovalis* fruits with the plants bearing them were collected together from Penghu Islands on July 9^th^, 2014. The floating duration was tested with the whole plants, since this is the likely mode when the seagrass is dislodged from sediment. The same types of containers described above were used.

### Floatation of seeds

The floatation duration of *T*. *hemprichii* seeds was tested in the same way described above between February 6 and 20, 2014. This was done by using seeds released in the fruit floatation experiment. The seeds of *T*. *hemprichii* are non-dormant, *i*.*e*., short sprouts could often be seen when freshly released from fruits. In this study, we defined those with more than one cm of leaves as germinating successfully, and thus became seedlings.

Floatation of *H*. *ovalis* seeds was tested the same way in July 2014, when fruits were readily available. Seeds of *H*. *ovalis* had hard seed coats. Thus, germination was easy to observe when the shoots protruded from the seed coats.

### Floatation of seedlings

After the seeds of *T*. *hemprichii* in the previous experiments germinated and became seedlings, most of them did not secure to the bottom since the plastic containers had no sand. As their leaves grew, the oxygen in the lacunar system accumulated[27]. Therefore, the seedlings became lighter than seawater. The dates on which each seedling started to float and later settled again were recorded to measure the duration of their floatation in the water column. Seedlings of *H*. *ovalis* never floated.

### Flotation of rhizome fragments

Dug-up plants were used in this experiment. We first used one-node rhizome fragments of both species, which contained leaves, stems and roots at the meristematic growth point. Due to the small sizes of *H*. *ovalis* fragments, three-node fragments containing three meristematic growth points and each with leaves, stems and roots, were also used. In the field experiment at Dongsha Island, 250 one-node rhizome fragments of each species, and 250 three-node fragments of *H*. *ovalis* were used on November 14, 2013. Fifty fragments were put into double layer laundry bags with mesh sizes of 5 mm and 2 mm. Bags was retrieved after 0, 1, 2, 4, 8 weeks to examine the conditions of these fragments. These bags were fixed in the sea with the help of breakwater blocks. After retrieval, they were kept in plastic containers to test for buoyancy and survival of each fragment.

For an indoor experiment, 100 two-node fragments of both *T*. *hemprichii* and *H*. *ovalis* were collected. They were kept in 45 x 30 cm plastic containers with aeration and water filtration. Buoyancy and survival of these fragments were checked at 0, 1, 2, 4, 8, 16 weeks. Live plants were returned to the containers for further observation. Dead *T*. *hemprichii* were dark and soft, often with leaves fallen off. Dead *H*. *ovalis* were dark, decomposing and also with fallen leaves.

### Birds as vectors

A duck (*Anas platyrhynchos*) and a goose (*Anser anser domesticus*) were used to test whether fruits and seeds of two species of seagrasses could pass through the digestive tracts of birds and remain competent to germinate. Fruits and seeds of *T*. *hemprichii* and *H*. *ovalis* were collected in February and July 2014, respectively. Five fruits of *T*. *hemprichii* were fed to a bird in the morning and five in the afternoon. Then, feces of birds were checked for viable seeds until no more could be found. The procedure was repeated five times using a total of 50 fruits. The seeds of *H*. *ovalis*, 0.5 to 1 mm in diameter [28] were much smaller than that of *T*. *hemprichii*, and a magnifying glass was needed for examination. A sieve with fine mesh size was also employed to facilitate the searching of small seeds. This was done daily after feeding birds with fruits and seeds.

The seeds of *H*. *ovalis* found in the feces were put in a tank with 60 ml of seawater, with unfed seeds as a control. The seawater in the containers was changed daily with a continuous white light (Philip Pleu 18 w) to induce germination.

### Fish as vectors

*Oreochromis mossambicus* (tilapia) was used as a representative of fishes to test whether fruits and seeds of seagrasses could pass through their digestive tracts undamaged. A total of 20 fruits and 20 seeds of *T*. *hemprichii*, and 50 fruits and 500 seeds of *H*. *ovalis* were prepared for feeding to two fishes. The experiments were conducted on March 30, 2014 for *T*. *hemprichii* and August 3, 2014 for *H*. *ovalis*. Fruits and seeds were mixed in shrimp meat before feeding to the fish. Particulate matter on the bottom of the fish tank was collected to examine for presence of seeds. The collected seeds, along with control seeds not having been fed to fishes, were tested for their ability to germinate.

## Results

### Floatation of seagrass fruits

**Fig 1:**
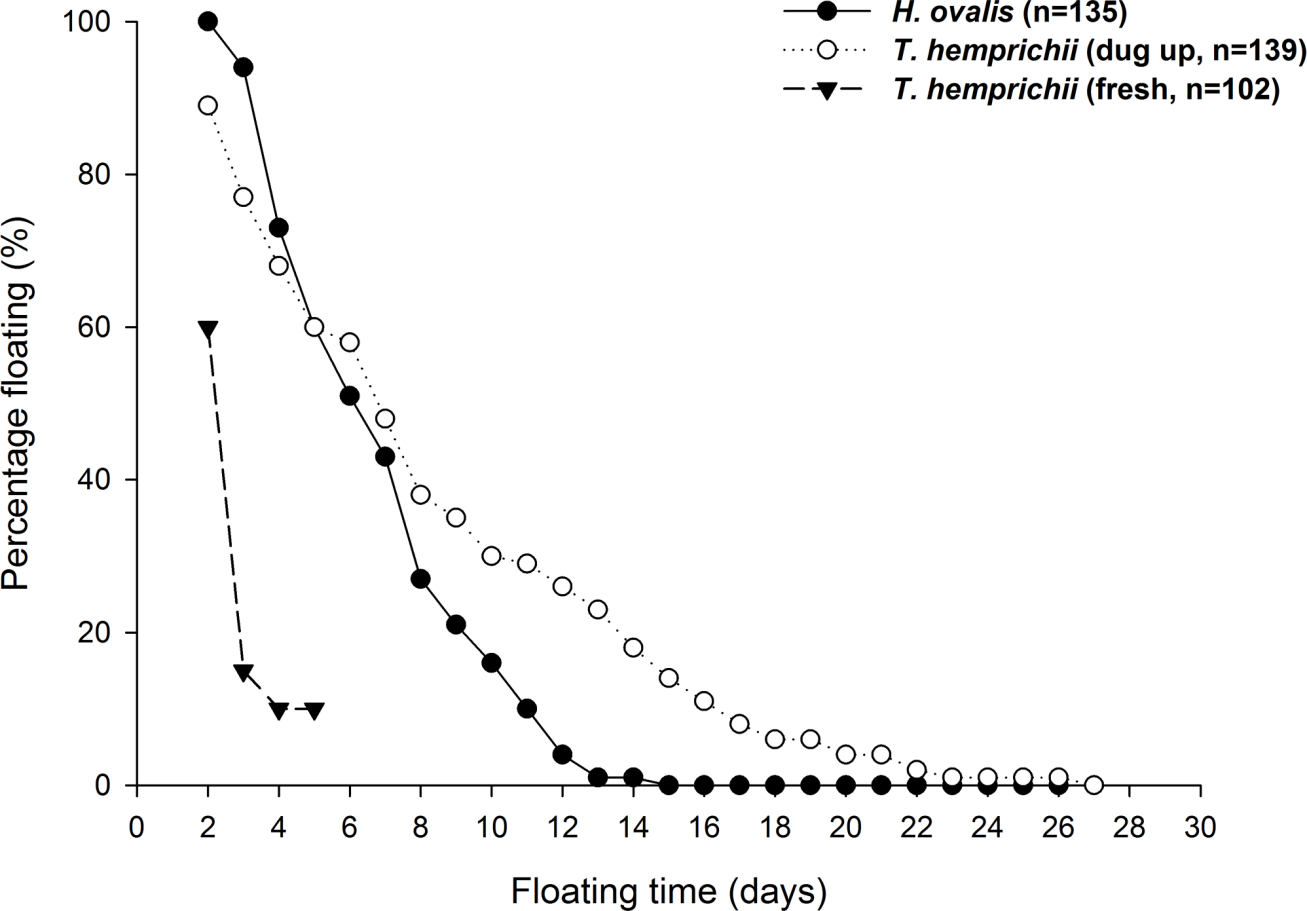
Flotation potential of *Thalassia hemprichii* and *Halophila ovalis* fruits. Percentages of fruits stay floating after collection from the beach.

The floatation potential, functioning as a dispersal mechanism, of *T*. *hemprichii* and *H*. *ovalis* fruits were examined (Fig 1). For the 102 fresh fruits in the first fruit floatation experiment, 85% dehisced in the first two days. The seeds were released and sank later. Five percent more dehisced in the third day, and no fruit dehisced in the fourth day. The observation stopped afterward. For the 10 fruits left intact, they seemed to be smaller than those dehisced. For the 139 fruits of *T*. *hemprichii* dug out from piles of seagrass debris in the second fruit floatation experiment, 38% and 14% remained floating after one and two weeks, respectively; the last one dehisced on day 26. For the 135 fruits of *H*. *ovalis*, 25% and 0% remained floating after one and two weeks, respectively (Fig 1).

**Fig 2:**
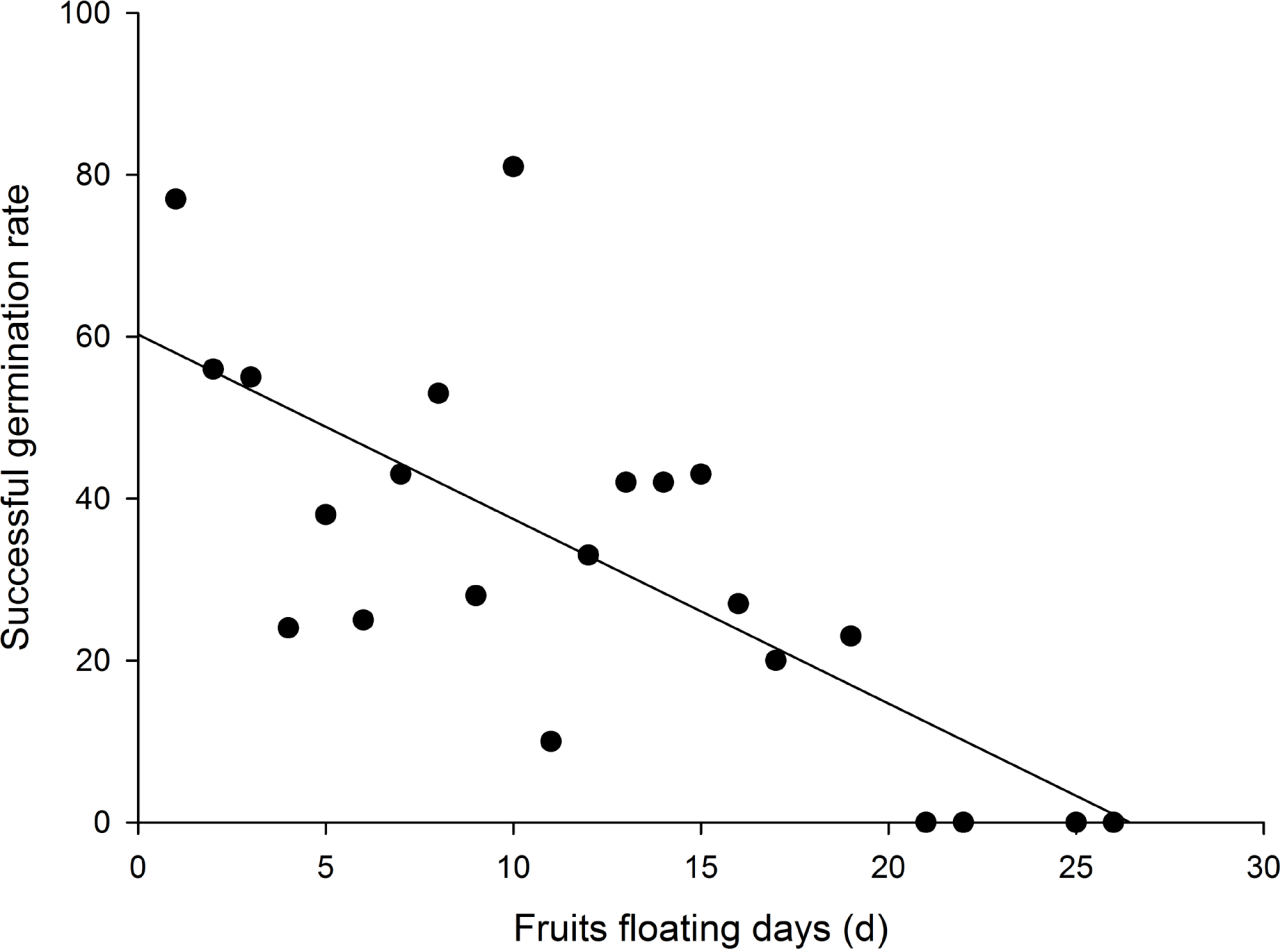
Thalassia hemprichii. Germination rates of seeds from dehiscing fruits of different floating days. N = 139. R^2^ = 0.53, Y = -2.27*X+60.2.

From those *T*. *hemprichii* fruits originally-buried in seagrass debris piles, a negative relationship was found between fruit floatation time and the germination rates of the seed they released (Fig 2). For example, 60% of these seeds germinated from those fruits floated for one day, but none of the seeds germinated from fruits floated for 20 days. No such experiment was tried on *H*. *ovalis*, since its seeds are known to have low germination rates.

### Floatation of seeds

**Fig 3:**
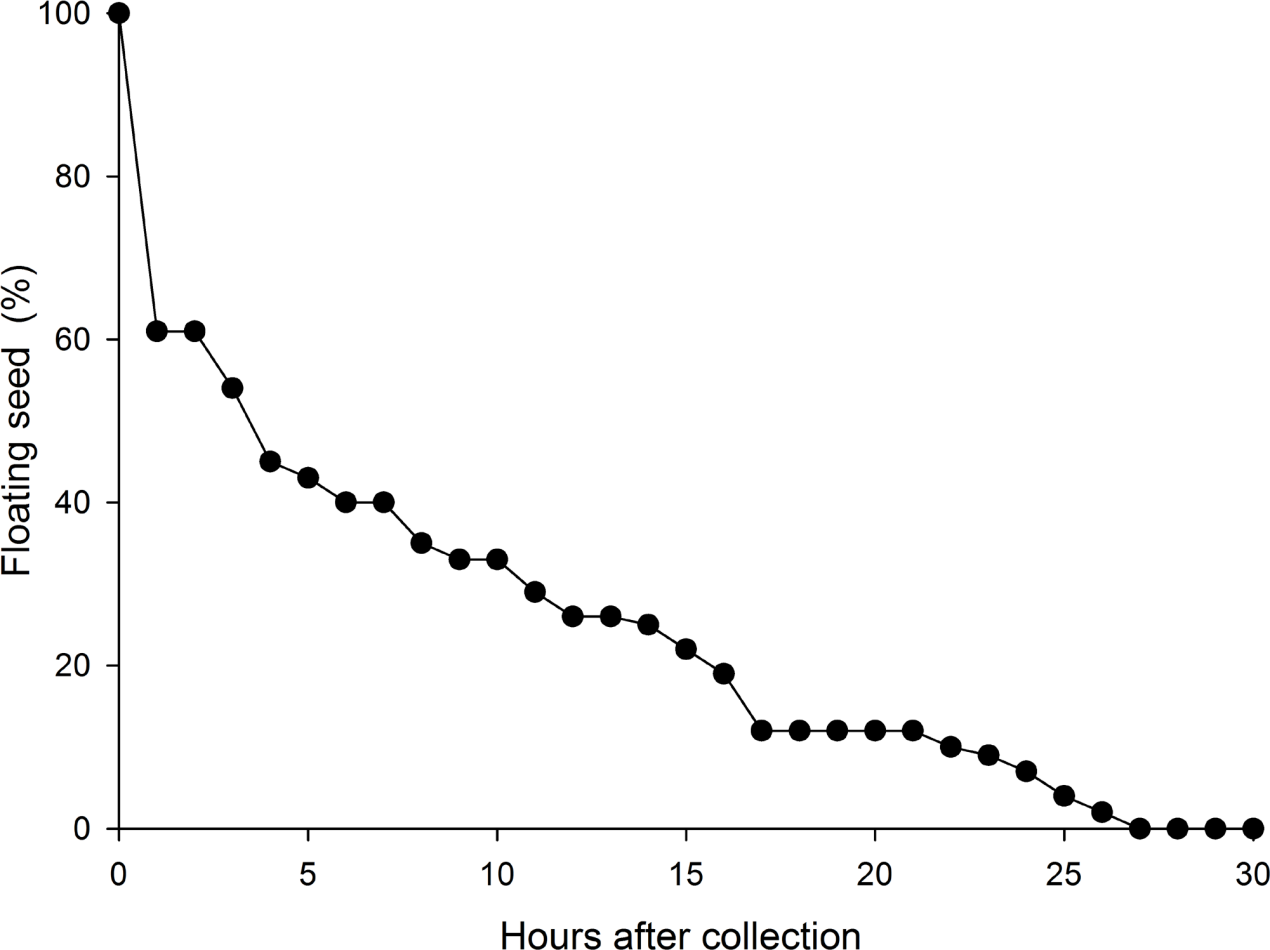
Thalassia hemprichii. Proportion of seeds floating after collection of fruits from beach. Starting n = 129.

The floating time of *T*. *hemprichii* seeds was short tested in 2011, but no detailed data was kept. In the experiment of 2014, the longest duration observed among 129 seeds was 26 hours in the laboratory (Fig 3). None of the 1200 seeds of *H*. *ovalis* could float; they sank immediately in the test.

### Floatation of seedlings

A total of 212 *T*. *hemprichii* seeds were used in the floatation/germination experiment. Thirty-two of the seeds grew leaves and became floating seedlings, presumably due to oxygen accumulated in the lacunar system. The durations of the seedling floatation in the laboratory was from 27 to 38 days, with an average of 32 days. Eventually, all seedlings of *T*. *hemprichii* sank to the bottom. The seedlings of the other species, *H*. *ovalis*, did not float at all.

### Floatation of rhizome fragments

**Fig 4:**
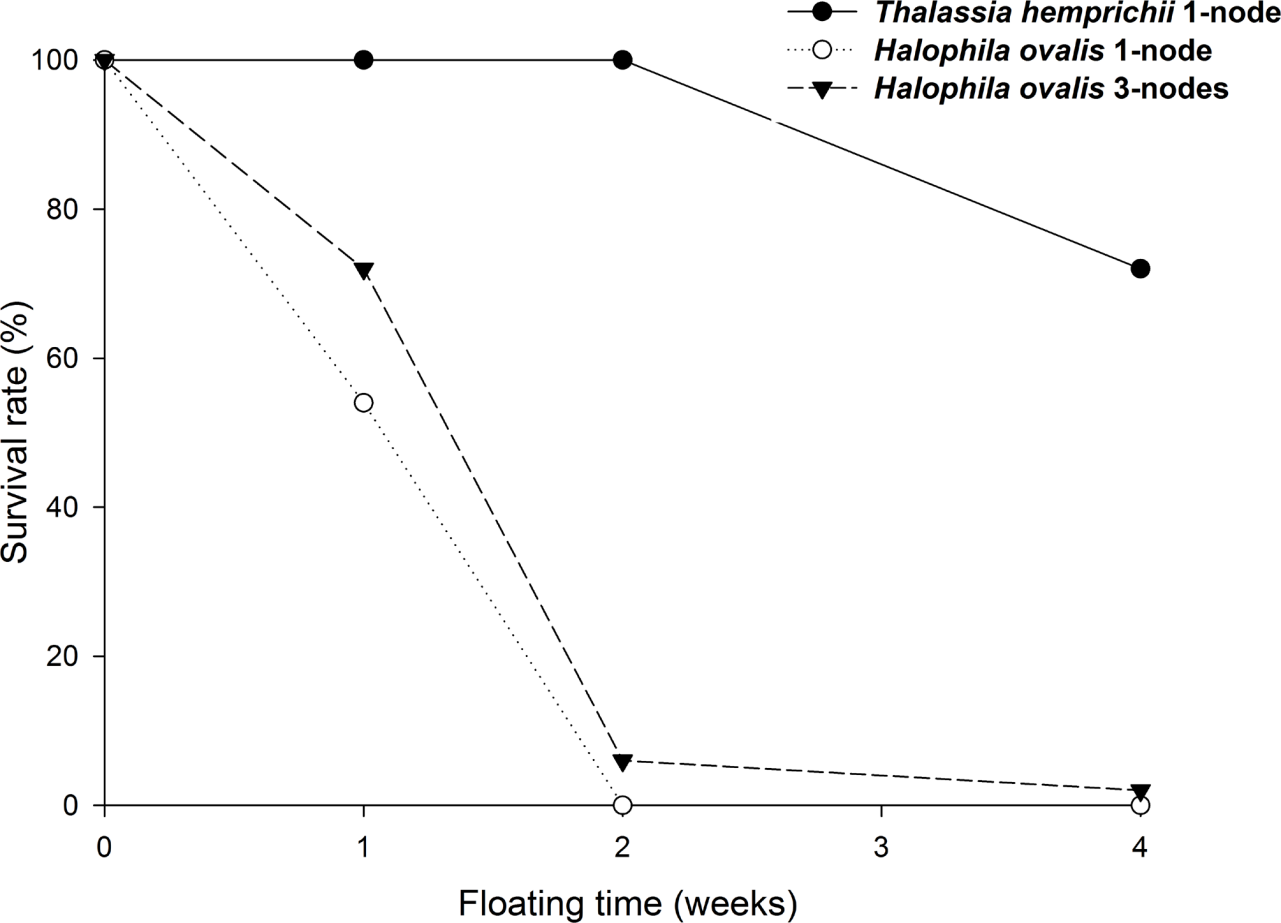
*Thalassia hemprichii* and *Halophila ovalis*. Survival rates of floating plants in the sea. Experiment duration: Nov. 14 to Dec. 12, 2013. Starting at n = 250 for each of the three groups. Survival rates are dependent on species since the end of first week (P<0.01, Fisher’s Exact Tests). Within *H*. *ovalis*, the survival is dependent on node number (P<0.01, in week 1 and 2, but P = 0.06 at week 4 when most were dead, Fisher’s Exact Tests).

In the outdoor experiment, 72% of one-node fragments of *T*. *hemprichii* rhizome survived and were buoyant after four weeks. In contrast, 0% of one-node and 2% of three-node fragments of *H*. *ovalis* rhizome stayed buoyant in the same period (Fig 4). Survival rates were dependent on species after the end of first week (P<0.01, Fisher’s Exact Tests); *T*. *hemprichii* survived better than *H*. *ovalis*. The non-buoyant *H*. *ovalis* lost their leaves and had darkened and soft stems; they were dead and decomposing. Within *H*. *ovalis*, the survival is dependent on node number (P<0.01, in week 1 and 2, but P = 0.06 at week 4 when most were dead, Fisher’s Exact Tests.); the 3-node fragments survived better than 1-node ones.

**Fig 5:**
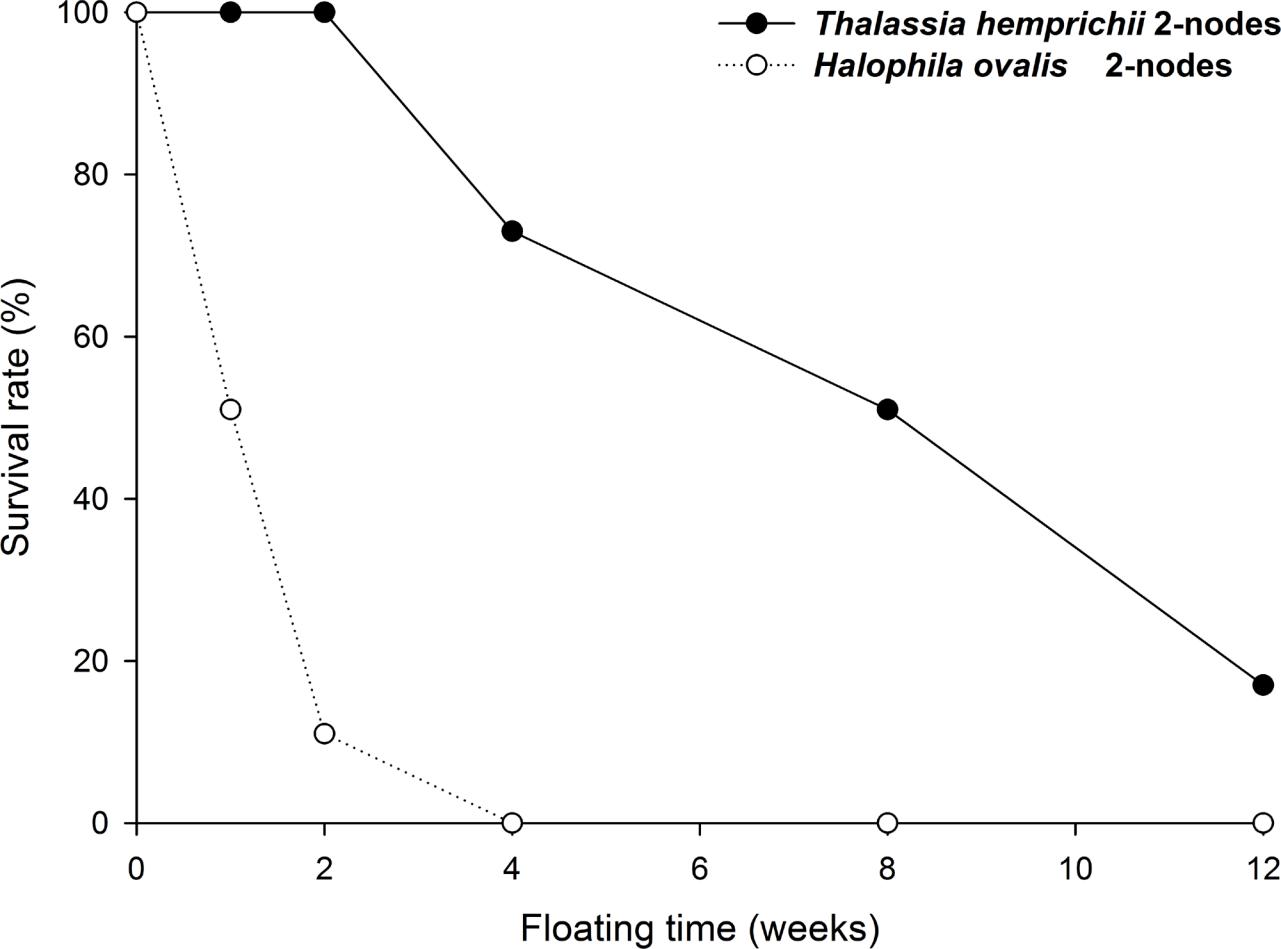
*Thalassia hemprichii* and *Halophila ovalis*. Survival rates of floating plants in laboratory. Starting with n = 100 for both species. Mortality rates are dependent on species since the end of the first week (P<0.01, Fisher’s Exact Tests.)

The indoor experiment on rhizome fragments was observed for three months. Among the two-node rhizome fragments, 73% of *T*. *hemprichii* and 0% of *H*. *ovalis* survived and remained floating after four weeks. The survival rates for *T*. *hemprichii* were 51% and 17% after eight and 12 weeks, respectively (Fig 5). Survival was dependent on species since the end of the first week (P<0.01, Fisher’s Exact Tests).

### Birds as vectors

**Fig 6:**
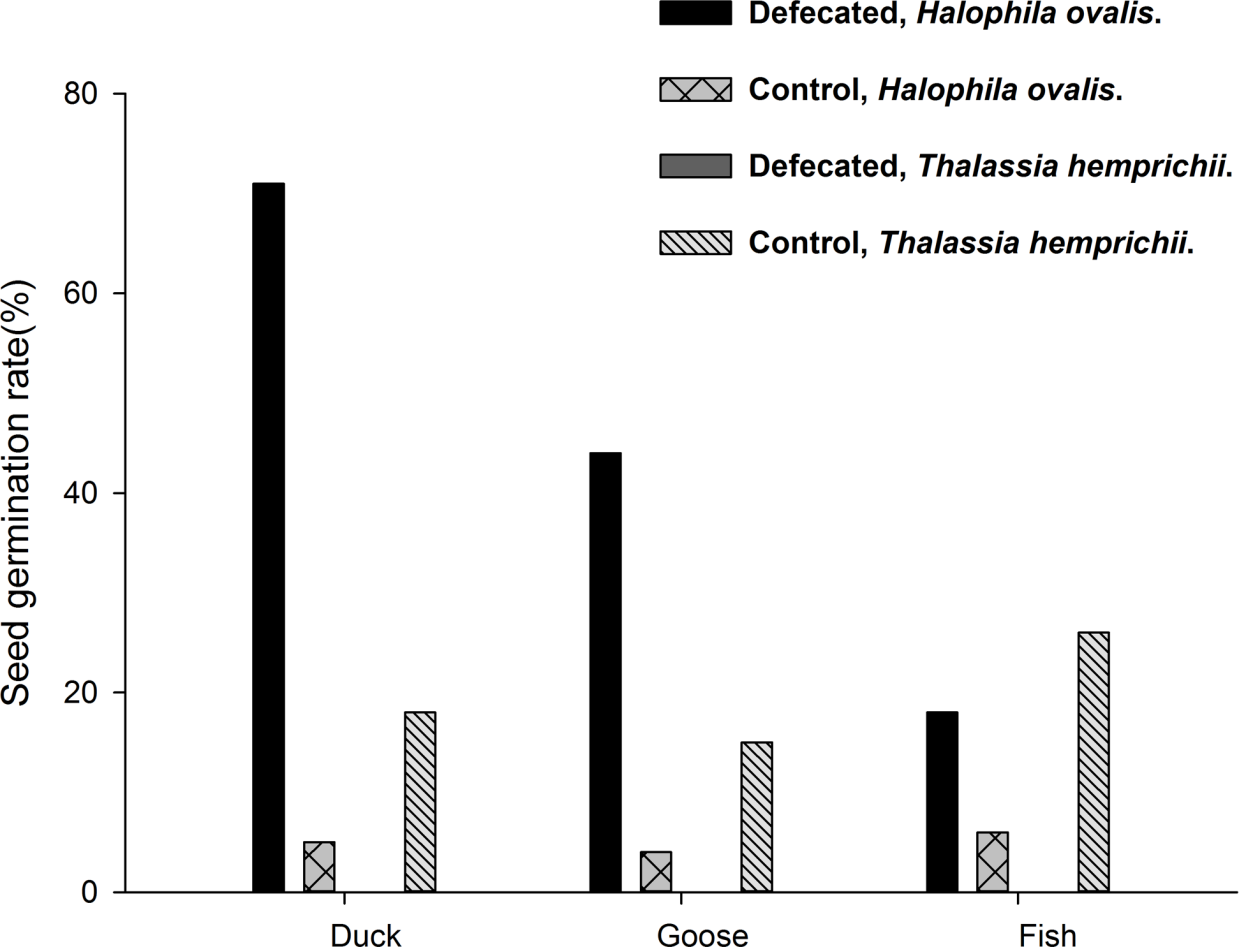
*Thalassia hemprichii* and *Halophila ovalis*. Seed germination rates in the feeding experiment of seagrass fruits. The X-axis indicates herbivores.

In the fruit feeding experiment, the feces of the birds were checked for seven consecutive days, but no intact seeds of *T*. *hemprichii* were ever found. The germination rates in the control group of seeds, without been ingested, were 17% (n = 100) (Fig 6).

In *T*. *hemprichii*, none of the seeds survive gut passage. In *H*. *ovalis*, seeds surviving gut passage had significantly higher germination rates in two birds than in control seeds but no such pattern occurred in the fish comparison (n = 360 in each comparison). Duck, 14 defecated, P<0.01; Goose, 16, defecated, P<0.01; fish, 11 defecated, P = 0.15; Fisher’s Exact Test.

A similar experiment, but using fruits of *H*. *ovalis*, was conducted in 2014. Out of 50 fruits fed to a duck, eight and six seeds were found in the 1^st^ and 2^nd^ day feces, but not any more in the five following days. Ten of these 14 seeds (71%) germinated successfully; the control group without going through guts had a 5% (n = 360) germination rate (P<0.01, Fisher’s Exact Test). For those fed to a goose, 11 and five seeds of *H*. *ovalis* were found in the 1^st^ and 2^nd^ day feces. Seven of these seeds (44%) germinated, compared to 4% (n = 360) for the control group (Fig 6, P<0.01, Fisher’s Exact Test).

**Fig 7:**
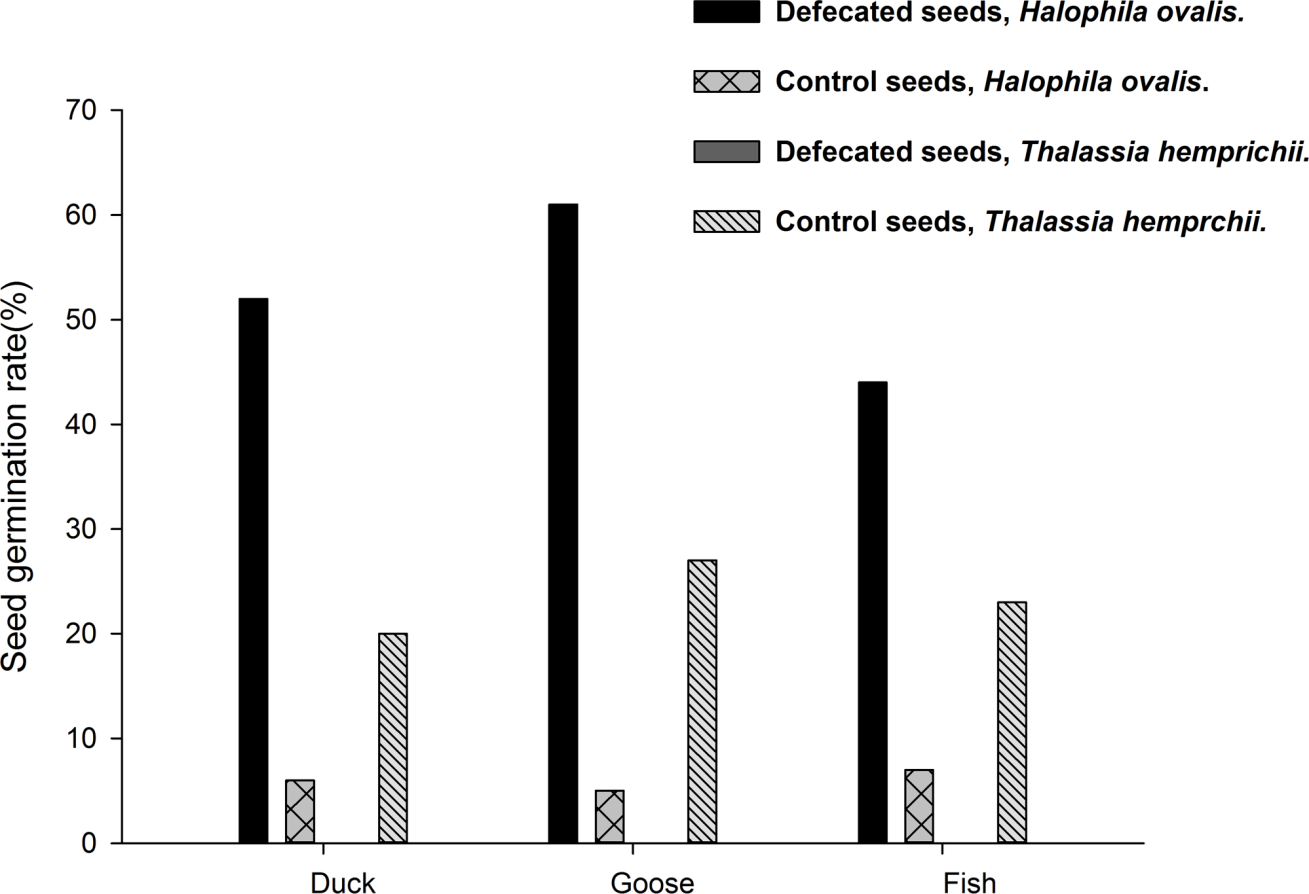
*Thalassia hemprichii* and *Halophila ovalis*. Seed germination rates in the feeding experiment of seagrass seeds. In *T*. *hemprichii*, none of the seeds survive gut passage. In *H*. *ovalis*, seeds surviving gut passage had significantly higher germination rates in two birds and a fish than in control seeds (n = 360 in each comparison). Duck, 27 defecated, P<0.01; Goose, 18 defecated, P<0.01; fish, 9 defecated, P<0.01; Fisher’s Exact Tests in all above.

In experiments where seeds were fed directly to the birds, no intact seed of *T*. *hemprichii* was found in the feces of the duck and the goose after feeding 50 seeds to each of them, in a total of five bouts (10 in each). The seeds in the control group had a germination rate of 20 and 27% (n = 40, 40) for the duck and the goose, respectively. For *H*. *ovalis*, 360 seeds were fed to the duck and the goose, respectively. Fourteen, nine and four seeds were found in the first three day duck feces, respectively, and no more were found in later days. These seeds passing the duck digestive tract had a germination rate of 52% (14/27); the control had a 6% (n = 360) germination rate (P<0.01, Fisher’s Exact Test). In the goose experiment, 12 and six seeds were found in the 1^st^ and 2^nd^ day feces. These defecated seeds had a 61% germination rate (11/18). The control seeds had a 5% (n = 360) germination rate (P<0.01, Fisher’s Exact Test, Fig 7).

### Fish as vectors

Ten fruits of *T*. *hemprichii* were fed to the fish; no intact seeds were found in the feces at the end of the experiment. The control had a 26% (n = 50) germination rate. In *H*. *ovalis*, 50 fruits were fed to the fish, and seven and four seeds were found in 1^st^ and 2^nd^ day feces. No more seeds were found in the following five days. The germination rate of these defecated seeds was 18% (2/11); the control seeds had a 6% (n = 360) germination rate (P = 0.15, Fisher’s Exact Test, Fig 6).

No intact seeds of *T*. *hemprichii* were found in the feces of the fish fed with 10 seeds in March 2014. The control seeds had a 24% (n = 80) germination rate. For *H*. *ovalis*, 360 seeds were fed to the fish, and six and three seeds were found in 1^st^ and 2^nd^ day feces, respectively. No more seeds were found in the following four days. A 44% (4/9) germination rate was found for these defecated seeds; the control had a 7% (n = 360) germination rate (P<0.01, Fisher’s Exact Test, Fig 7).

## Discussion

The two seagrasses studied here obviously accomplish long-distance dispersal by different mechanisms at different life history stages, with neither of them relying on floating seeds.

The turtle grass, *T*. *hemprichii*, disperses by floating on the sea surface. The adult plants, in the form of rhizome fragments [10] have the greatest potential to float for a long time, and thus potentially travel a long distance while maintaining the capacity to re-colonize new habitats. Their seedlings, when floating, may play a minor role due to their relatively shorter duration of floatation. The fruits we collected at two different environments had different floatation periods. Most fruits might dehisce and release seeds in a few days. They do not have the potential to travel far. Those buried in seagrass debris could travel on the sea surface longer, but suffer from low germination rates. The role of *T*. *hemprichii* seeds in long distance dispersal must be minor.

In Penghu Islands in Taiwan Strait, where *T*. *hemprichii* was never reported by seagrass researchers, floating plants of the species near the coast were noticed[29]. This suggests that adult plants of *T*. *hemprichii* could indeed travel long distances. The researchers planted these floating *T*. *hemprichii* collected from waters at Penghu, and found a ~20% successful rate in non-carbonate sediment after a 3-month duration [30].This result is a good indication that rhizome fragments of *T*. *hemprichii*, after traveling long distance on sea surface, could remain competent to start a new life in a distant habitat.

The plants of shrimp grass, *H*. *ovalis*, on the other hand, do not have the capacity to float for much time; they are more likely to disperse by the small and resistant seeds after been ingested by birds, for a fast but potentially long-distance journey in the air. Although *H*. *ovalis* seeds could also pass through the guts of fishes successfully, the distance traveled must be much shorter than that by birds. This notion is supported by the observation that all birds, except a waterhen, *Amaurornis phoenicurus*, are migratory at Dongsha [31]. They are highly mobile and are presumably good vectors between habitats on different islands. Most fishes around seagrass beds, on the other hand, are small, benthic fishes [32].

Three of the four life history stages of *T*. *hemprichii* have floating ability. For example, the fresh fruits tested in this survey floated an average of about 1–2 days, and about 5 days for buried fruits, if they are resuspended. It was suggested that floating fruits/seeds were capable of traveling 10’s of kilometers under extreme (typhoon) and consistent current conditions. The point of argument here is that under the same condition, rhizome fragments could stay on the seawater surface for a much longer duration, and thus have greater potential to disperse farther.

One discovery in this study is the correlation between floating days and germination rates in dup-up seeds of *T*. *hemprichii* (Fig 2). We suggest this is an indication that these seeds are not adaptive for long distance dispersal. The floatation days recorded in our experiment could not really be interpreted as meaningful dispersal days at sea, since seeds lost their viability during the process. Like the Quezon study in the Philippines [14], most free fruits of *T*. *hemprichii* on the beach released seeds in a few days once in water again. In fact, most fruits (90%) of *T*. *hemprichii* may dehisce before detaching from mother plants [33]; these released seeds must be scattered near natal habitats. The remaining seeds are carried by floating fruits for a journey of variable duration. Obviously, fruits and seeds of *T*. *hemprichii* do not float long and are not an adaptive stage for long distance dispersal.

In a remote island like Dongsha where populations are more or less isolated from others, the long distance dispersal capability, *e*.*g*., floating ability, could not be a result of local selection. This is because successful long-distance travelers end up elsewhere, and have no contribution to local gene pools. In other words, favorable currents, such as during monsoons, could not be selective forces for long-distance dispersal, but are coincidental or accidental in contributing to patterns of geographical distribution.

The seeds of *T*. *hemprichii* have several adaptations to help them secure to substrate of carbonate sand [17]. Obviously, fixing to the bottom is more important a function than dispersing, and the two are contradictory. Once failing to attach to bottom, *e*.*g*., when the densities of seagrasses on the bottom are high, or when the substrate is comprised of silicate sand, the growing seedlings could not secure to the bottom. However, with air in the lacunar system, they could float to water surface. The discovery in this study is that the seedlings were able to float for about 30 days, and then sink again. During the floatation period, they could be carried by current and disperse far from natal habitats. These seedlings are more important a stage than fruits/seeds for dispersal, since they obviously stay for a longer time near the water surface. Due to their relatively small size, the seedlings are likely to be lodged in zones of coral fragments (Soong, personal observation) often found in shallow waters, *e*.*g*., backreefs behind reef crests at Dongsha Island. It is uncertain how important this stage is for long distance dispersal, relative to rhizome fragments of adults, given the 30 days floatation duration. External factors such as seasonal wind, current, could affect the speed and direction of the travel [33](Tables 1 and 2).

**Table 1:** Thalassia hemprichii. Comparison of dispersal related traits at various life history stages.

Characteristics	Life history stages		
	Fruits/seeds	Seedlings	Rhizome fragments
Season of production	Fruiting season, Feb-Mar at Dongsha	After fruiting season, Feb-April	Monsoons and typhoons (Oct-Feb, and Jun-Sep at Dongsha)
Distance dispersed	Short	Intermediate	Long
Condition of production	Fruiting	Failure to secure to substrate, due to high local density, inadequate substrate	Disturbance
Dependence on lacunars	No	Yes	Yes
New adaptation to dispersal	Fast securing to substrate	Not known	Not known
Potential landing sites	Subtidal/ intertidal	Subtidal/intertidal	intertidal
Development origin	Sexual	Sexual	Asexual

**Table 2:** Halophila ovalis. Comparison of dispersal related traits at various life history stages.

Characteristics	Life history stages		
	Fruits	Seeds	Rhizome fragments
Season of production	Fruiting season unclear.	Seed banks exist a long time.	Monsoons and typhoons (Oct-Feb, and Jun-Sep, at Dongsha.)
Distance dispersed	Not known, they are buried in sediment.	Long, if by bird vectors. Short, been moved on sediment surface.	Short, been moved on sediment.
Condition of production	Fruiting.	Fruiting, suspension by disturbance.	Disturbance.
Dependence on lacunars	No	No	Yes
New adaptation to dispersal	Not known	Not known	Not known
Potential landing sites	Subtidal/ Intertidal	Subtidal/ Intertidal where migrating birds visit	Intertidal
Development origin	Sexual	Sexual	Asexual

Adult plants of *T*. *hemprichii* could become dislodged from the substrate by breaking waves during winter monsoons or summer typhoons (Authors’ personal observation at Dongsha Island). They usually occur in floating masses reaching 40 cm across, and often with other species, *e*.*g*., *Syringodium isoetifolium*. Presumably, they are also able to float for a long time, if not intercepted by beaches, and be carried by currents for a long distance. In our tests, bare stems could grow new roots as long as they remained hard. Thus, the adult plant stage of *T*. *hemprichii* could serve as propagules and disperse effectively. This dispersal by asexual means is also reported in other seagrasses [34,35].

The production seasons of rhizome fragments may vary depending on regions. At Dongsha, the production of these dispersal units is temporally more spread than the floating seedlings which only occur right after fruiting seasons, *i*.*e*., February to April. Despite lots of examples of morphological adaptations after colonization of marine environments by seagrasses [12], the long-distance dispersal of *T*. *hemprichii* does not seem to involve any of these newly acquired traits. The break-off of rhizome fragments does not seem aided by any special characteristics, *e*.*g*., constrictions of stems. New adaptation for dispersal at the seedling stage was not found as floatation of seedlings is a necessary result of failure to secure to substrate; accumulation of air in the lacunar sooner or later makes the whole plants lighter than seawater.

*Halophila ovalis* has very limited capacity to disperse by sea, as indicated by the short duration of various life history stages that remain floating and alive (Table 2). Larger fragments, *e*.*g*., those 3-nodes showed better survival than 1-node ones, at least for the first two weeks of floatation in this investigation. Similar size-correlated fragment survival rates occurred in other marine organisms capable of vegetative propagation, *e*.*g*., corals [36], Thus, there is a reason to suspect that if the floating mass is much larger, the survival could improve. An experiment involving a large mass of *H*. *ovalis* is not practical at Dongsha due to limited availability. The seeds of *H*. *ovalis*, however, are obviously resistant and could survive the digestive tracts of birds and fishes as revealed in this investigation. Since birds could move fast, one to two days residence of seeds in bird guts could potentially disperse the seeds hundreds of kilometers [9,10,37]. This is much greater than movement by bulk sediment transport [38] or by non-migratory animals [39–41], in both spatial scale and in frequency. Migratory birds generally move in north-south directions; the dispersal by these vectors are necessarily limited to these directions. Whether birds are likely to ingest fruits or seeds of *H*. *ovalis* remain difficult to test. Genetic studies could be used to test this bird-vector hypothesis that predicts populations distributed in east-west direction are more likely to be segregated if migrating birds are the major agents of dispersal.

The same residence time, but in fish guts, could result in only relatively limited ranges of dispersal for two reasons. In addition to much slower speed, benthic animals may be less likely to travel between discrete islands separated by deep water. One to two days in guts are still enough to spread the seagrasses locally, as their seeds are heavier than water and could not be moved much on sediment by water motions. In comparison, ingestion by birds is a more likely mechanism for long-distance dispersal.

In our experiment, most seeds of *H*. *ovalis* could not be found after fed to birds or fishes. They were likely ground and digested by the animals. In other words, only a small percentage of ingested seeds pass through digestive systems of the animals unharmed. Some seeds of *H*. *ovalis* did pass fish/bird guts successfully, and enjoyed much higher germination rates than the controls without going through animal guts. This is a temporal advantage, since dormant seeds of *H*. *ovalis* may germinate later. This phenomenon suggests that some inhibitory factors delaying germination of seeds might be removed during the gut passage. These same factors may have kept *Halophila* seeds dormant in the sediment [26]. In fact, the ability of *H*. *ovalis* seeds to pass through digestive tracts of animals unharmed may be a side-effect of its capability of long-term dormancy in the sediment. The thick and rugged seed coats might protect the seeds from adverse elements in both the sediments and the digestive tracts [42].

The small and dormant seeds of *H*. *ovalis*, on the other hand, may still be relied upon for dispersal as their terrestrial ancestors do. Their small size allows them to be transported easily by physical forces within reefs, as long as they stay in relatively shallow waters. Small seed sizes also mean that they may be ingested accidentally, rather than as food [40]. Their protective and resistant coatings, presumably adaptive for dormancy when buried in substrate, now potentially allow them to be carried in guts of flying vectors to disperse to far away locations. Due to the fragility of adult plants once dislodged from habitats, the seeds of *H*. *ovalis* seem to be the only stage in its life history capable of dispersing to disjunctive habitats.

For most terrestrial plants, seeds are the default dispersal stage. Most terrestrial seeds sink in seawater, as are the cases of the two seagrasses of this study. An alternative dispersal mechanism is needed when a grass evolves to become a seagrass. In the two species studied here, different methods of dispersal were adopted, or rather, happened to work. Traits, *i*.*e*., oxygen-filled lacunar in *T*. *hemprichii*, and bird-carrying capability in *H*. *ovalis*, must have existed even before the two became marine species. These “pre-adapted” traits allowed the seagrasses to expand territory in the sea. This is important because local events would not wipe out the entire species once the distribution of the species became widespread.

## Supporting Information

S1 Table *Thalassia hemprichii* and *Halophila ovalis*. Survival (floating) numbers in the Floatation Experiment at Dongsha Island (Field).
(DOCX)

S2 Table *Thalassia hemprichi*i and *Halophila ovalis*. Survival (floating) numbers in the Floatation Experiment (Lab).
(DOCX)

S3 Table Thalassia hemprichii. Germination rates after floating for various days.
(DOCX)

S4 Table *Thalassia hemprichii* and *Halophila ovalis*. Germination rates in feeding expt.
(DOCX)

S5 Table Halophila ovalis. Fruit floatation experiment in lab.
(DOCX)

S6 Table Thalassia hemprichii. Seedling floatation experiment in lab. Beginning n = 32.
(DOCX)
