# Supplementary material for: Long Distance Dispersal Potential of Two Seagrasses Thalassia hemprichii and Halophila ovalis
Source: PLoS One. 2016 Jun 1;11(6):e0156585. doi: 10.1371/journal.pone.0156585 (PMC4889049; doi:10.1371/journal.pone.0156585)
Supplement: S1 Table — Survival (floating) numbers in the Floatation Experiment at Dongsha Island (Field). (DOCX) [file pone.0156585.s001.docx]

**S1 Table. *Thalassia hemprichii* and *Halophila ovalis*. Survival (floating) numbers in the Floatation Experiment at Dongsha Island (Field)**

| Week | *T. hemprichii*. 1-node | *H.ovalis*, 1-node | *H. ovalis*, 3-node |
| --- | --- | --- | --- |
| 0 | 250 | 250 | 250 |
| 1 | 250 | 135 | 180 |
| 2 | 250 | 0 | 15 |
| 4 | 180 | 0 | 5 |
