# Supplementary material for: Long Distance Dispersal Potential of Two Seagrasses Thalassia hemprichii and Halophila ovalis
Source: PLoS One. 2016 Jun 1;11(6):e0156585. doi: 10.1371/journal.pone.0156585 (PMC4889049; doi:10.1371/journal.pone.0156585)
Supplement: S2 Table — Survival (floating) numbers in the Floatation Experiment (Lab). (DOCX) [file pone.0156585.s002.docx]

**S2 Table. *Thalassia hemprichi*i and *Halophila ovalis.* Survival (floating) numbers in the Floatation Experiment (Lab)**

| Week | *T. hemprichii,* 2-node | *H. ovalis*, 2-node |
| --- | --- | --- |
| 0 | 100 | 100 |
| 1 | 100 | 51 |
| 2 | 100 | 11 |
| 4 | 73 | 0 |
| 8 | 51 | 0 |
| 12 | 17 | 0 |
