# Supplementary material for: Long Distance Dispersal Potential of Two Seagrasses Thalassia hemprichii and Halophila ovalis
Source: PLoS One. 2016 Jun 1;11(6):e0156585. doi: 10.1371/journal.pone.0156585 (PMC4889049; doi:10.1371/journal.pone.0156585)
Supplement: S3 Table — Germination rates after floating for various days. (DOCX) [file pone.0156585.s003.docx]

**S3 Table *Thalassia hemprichii*. Germination rates after floating for various days**

| Fruit floating days | Number of fruits | Number of seeds released | Number of seeds germinated | Number of dead seeds | Germination rates after x days of floating | % of fruits floating |
| --- | --- | --- | --- | --- | --- | --- |
| Day1 | 15 | 60 | 46 | 14 | 77% | 89% |
| Day 2 | 17 | 68 | 38 | 30 | 56% | 77% |
| Day 3 | 13 | 49 | 27 | 23 | 55% | 68% |
| Day 4 | 11 | 42 | 10 | 32 | 24% | 60% |
| Day 5 | 3 | 13 | 5 | 8 | 38% | 58% |
| Day 6 | 13 | 53 | 13 | 40 | 25% | 48% |
| Day 7 | 14 | 54 | 23 | 31 | 43% | 38% |
| Day 8 | 5 | 19 | 10 | 9 | 53% | 35% |
| Day 9 | 6 | 25 | 7 | 18 | 28% | 30% |
| Day 10 | 1 | 3 | 2 | 1 | 81% | 29% |
| Day 11 | 5 | 18 | 2 | 16 | 10% | 26% |
| Day 12 | 4 | 15 | 5 | 10 | 33% | 23% |
| Day 13 | 7 | 26 | 11 | 15 | 42% | 18% |
| Day 14 | 6 | 26 | 11 | 15 | 42% | 14% |
| Day 15 | 4 | 14 | 6 | 8 | 43% | 11% |
| Day 16 | 4 | 15 | 4 | 11 | 27% | 8% |
| Day 17 | 3 | 10 | 2 | 8 | 20% | 6% |
| Day 18 | None | none | none | none | none | 6% |
| Day 19 | 3 | 13 | 3 | 10 | 23% | 4% |
| Day 20 | None | none | none | none | none | 4% |
| Day 21 | 2 | 9 | 1 | 8 | 0% | 2% |
| Day 22 | 1 | 5 | 1 | 4 | 0% | 1% |
| Day 23 | None | none | none | none | none | 1% |
| Day 24 | None | none | none | none | none | 1% |
| Day 25 | 1 | 4 | 0 | 4 | 0% | 1% |
| Day26 | 1 | 4 | 0 | 4 | 0% | 0% |
