# Supplementary material for: Long Distance Dispersal Potential of Two Seagrasses Thalassia hemprichii and Halophila ovalis
Source: PLoS One. 2016 Jun 1;11(6):e0156585. doi: 10.1371/journal.pone.0156585 (PMC4889049; doi:10.1371/journal.pone.0156585)
Supplement: S4 Table — Germination rates in feeding expt. (DOCX) [file pone.0156585.s004.docx]

**S4 Table *Thalassia hemprichii* and *Halophila ovalis*. Germination rates in feeding expt.**

| Animal,  Seagrass,  Fruit/seed | Number fed | Defecated 1^st^ day | Defecated 2^nd^ day | Defecated, 3^rd^ day | No. germinated | Germination Rate (GR) | G R in control |
| --- | --- | --- | --- | --- | --- | --- | --- |
| Duck, H.o., seed | 360 | 14 | 9 | 4 | 14 | 52% | 6% |
| Goose  H.o. seed | 360 | 12 | 6 | 0 | 11 | 61% | 5% |
| Fish,,H.o., seed | 360 | 6 | 3 | 0 | 4 | 44% | 7% |
| Duck, T.h., seed | 50 | 0 | 0 | 0 | 0 | 0 | 20% |
| Goose, T.h., seed | 50 | 0 | 0 | 0 | 0 | 0 | 27% |
| Seed, T.h., seed | 10 | 0 | 0 | 0 | 0 | 0 | 23% |
| Duck, H.o., fruit | 50 | 8 | 6 | 0 | 10 | 71% | 5% |
| Goose, H.o., fruit | 50 | 11 | 5 | 0 | 7 | 44% | 4% |
| Fish, H.o., fruit | 50 | 7 | 4 | 0 | 2 | 18% | 6% |
| Duck, T.h., fruit | 50 | 0 | 0 | 0 | 0 | 0 | 18% |
| Goose, T.h., fruit | 50 | 0 | 0 | 0 | 0 | 0 | 15% |
| Fish, T.h., fruit | 10 | 0 | 0 | 0 | 0 | 0 | 26% |
