# Supplementary material for: Long Distance Dispersal Potential of Two Seagrasses Thalassia hemprichii and Halophila ovalis
Source: PLoS One. 2016 Jun 1;11(6):e0156585. doi: 10.1371/journal.pone.0156585 (PMC4889049; doi:10.1371/journal.pone.0156585)
Supplement: S5 Table — Fruit floatation experiment in lab. (DOCX) [file pone.0156585.s005.docx]

**S5 Table. *Halophila ovalis*. Fruit floatation experiment in lab**

| Date (2014) | Day | Floating number of plants | **%** |
| --- | --- | --- | --- |
| 7/9 | Day1 | 135 | 100% |
| 7/10 | Day2 | 127 | 94% |
| 7/11 | Day3 | 99 | 73% |
| 7/12 | Day4 | 81 | 60% |
| 7/13 | Day5 | 69 | 51% |
| 7/14 | Day6 | 58 | 43% |
| 7/15 | Day7 | 36 | 27% |
| 7/16 | Day8 | 29 | 21% |
| 7/17 | Day9 | 21 | 16% |
| 7/18 | Day10 | 13 | 10% |
| 7/19 | Day11 | 5 | 4% |
| 7/20 | Day12 | 2 | 1% |
| 7/21 | Day13 | 1 | 1% |
| 7/22 | Day14 | 0 | 0% |
