# Supplementary material for: Long Distance Dispersal Potential of Two Seagrasses Thalassia hemprichii and Halophila ovalis
Source: PLoS One. 2016 Jun 1;11(6):e0156585. doi: 10.1371/journal.pone.0156585 (PMC4889049; doi:10.1371/journal.pone.0156585)
Supplement: S6 Table — Seedling floatation experiment in lab. Beginning n = 32. (DOCX) [file pone.0156585.s006.docx]

**S6 Table. *Thalassia hemprichii*. Seedling floatation experiment in lab. Beginning n=32**

| No. | Starting date of floating | Date of sinking again | Days floating |
| --- | --- | --- | --- |
| 1. | 2014 5 1 | 2014 5 29 | 28 |
| 2. | 2014 5 1 | 2014 5 31 | 30 |
| 3. | 2014 5 1 | 2014 6 1 | 31 |
| 4 | 2014 5 2 | 2014 6 5 | 34 |
| 5 | 2014 5 2 | 2014 5 29 | 27 |
| 6 | 2014 5 2 | 2014 6 8 | 37 |
| 7 | 2014 5 2 | 2014 6 5 | 34 |
| 8 | 2014 5 3 | 2014 5 31 | 28 |
| 9 | 2014 5 3 | 2014 6 6 | 34 |
| 10 | 2014 5 5 | 2014 6 11 | 37 |
| 11 | 2014 5 7 | 2014 6 7 | 31 |
| 12 | 2014 5 7 | 2014 6 3 | 27 |
| 13 | 2014 5 7 | 2014 6 10 | 34 |
| 14 | 2014 5 7 | 2014 6 16 | 40 |
| 15 | 2014 5 7 | 2014 6 12 | 36 |
| 16 | 2014 5 8 | 2014 6 15 | 38 |
| 17 | 2014 5 9 | 2014 6 15 | 37 |
| 18 | 2014 5 11 | 2014 6 17 | 37 |
| 19 | 2014 5 11 | 2014 6 15 | 35 |
| 20 | 2014 5 12 | 2014 6 14 | 33 |
| 21 | 2014 5 12 | 2014 6 15 | 34 |
| 22 | 2014 5 13 | 2014 6 13 | 31 |
| 23 | 2014 5 15 | 2014 6 19 | 35 |
| 24 | 2014 5 15 | 2014 6 15 | 31 |
| 25 | 2014 5 15 | 2014 6 15 | 31 |
| 26 | 2014 5 15 | 2014 6 11 | 27 |
| 27 | 2014 5 16 | 2014 6 15 | 30 |
| 28 | 2014 5 17 | 2014 6 15 | 29 |
| 29 | 2014 5 18 | 2014 6 17 | 30 |
| 30 | 2014 5 19 | 2014 6 15 | 27 |
| 31 | 2014 5 19 | 2014 6 18 | 30 |
| 32 | 2014 5 19 | 2014 6 19 | 31 |

| No. | Starting date of floating | Date of sinking again | Days floating |
| --- | --- | --- | --- |
| 1. | 2014 5 1 | 2014 5 29 | 28 |
| 2. | 2014 5 1 | 2014 5 31 | 30 |
| 3. | 2014 5 1 | 2014 6 1 | 31 |
| 4 | 2014 5 2 | 2014 6 5 | 34 |
| 5 | 2014 5 2 | 2014 5 29 | 27 |
| 6 | 2014 5 2 | 2014 6 8 | 37 |
| 7 | 2014 5 2 | 2014 6 5 | 34 |
| 8 | 2014 5 3 | 2014 5 31 | 28 |
| 9 | 2014 5 3 | 2014 6 6 | 34 |
| 10 | 2014 5 5 | 2014 6 11 | 37 |
| 11 | 2014 5 7 | 2014 6 7 | 31 |
| 12 | 2014 5 7 | 2014 6 3 | 27 |
| 13 | 2014 5 7 | 2014 6 10 | 34 |
| 14 | 2014 5 7 | 2014 6 16 | 40 |
| 15 | 2014 5 7 | 2014 6 12 | 36 |
| 16 | 2014 5 8 | 2014 6 15 | 38 |
| 17 | 2014 5 9 | 2014 6 15 | 37 |
| 18 | 2014 5 11 | 2014 6 17 | 37 |
| 19 | 2014 5 11 | 2014 6 15 | 35 |
| 20 | 2014 5 12 | 2014 6 14 | 33 |
| 21 | 2014 5 12 | 2014 6 15 | 34 |
| 22 | 2014 5 13 | 2014 6 13 | 31 |
| 23 | 2014 5 15 | 2014 6 19 | 35 |
| 24 | 2014 5 15 | 2014 6 15 | 31 |
| 25 | 2014 5 15 | 2014 6 15 | 31 |
| 26 | 2014 5 15 | 2014 6 11 | 27 |
| 27 | 2014 5 16 | 2014 6 15 | 30 |
| 28 | 2014 5 17 | 2014 6 15 | 29 |
| 29 | 2014 5 18 | 2014 6 17 | 30 |
| 30 | 2014 5 19 | 2014 6 15 | 27 |
| 31 | 2014 5 19 | 2014 6 18 | 30 |
| 32 | 2014 5 19 | 2014 6 19 | 31 |
